# Neoplastic Transformation of T Lymphocytes through Transgenic Expression of a Virus Host Modification Protein

**DOI:** 10.1371/journal.pone.0034140

**Published:** 2012-04-27

**Authors:** Sílvia Cristina Paiva Almeida, Vivian Leite de Oliveira, Sónia Ventura, Margarita Bofill, Robert Michael Evans Parkhouse

**Affiliations:** 1 Instituto Gulbenkian de Ciência, Oeiras, Portugal; 2 Institucio Catalana de Recerca i Estudis Avancats, IrsiCaixa, Barcelona, Spain; Institut Pasteur, France

## Abstract

Virus host evasion genes are ready-made tools for gene manipulation and therapy. In this work we have assessed the impact *in vivo* of the evasion gene A238L of the African Swine Fever Virus, a gene which inhibits transcription mediated by both NF-κB and NFAT. The A238L gene has been selectively expressed in mouse T lymphocytes using tissue specific promoter, enhancer and locus control region sequences for CD2. The resulting two independently derived transgenic mice expressed the transgene and developed a metastasic, angiogenic and transplantable CD4^+^CD8^+^CD69^–^ lymphoma. The CD4^+^CD8^+^CD69^–^ cells also grew vigorously *in vitro*. The absence of CD69 from the tumour cells suggests that they were derived from T cells at a stage prior to positive selection. In contrast, transgenic mice similarly expressing a mutant A238L, solely inhibiting transcription mediated by NF-κB, were indistinguishable from wild type mice. Expression of Rag1, Rag2, TCRβ-V8.2, CD25, FoxP3, Bcl3, Bcl2 l14, Myc, IL-2, NFAT1 and Itk, by purified CD4^+^CD8^+^CD69^–^ thymocytes from A238L transgenic mice was consistent with the phenotype. Similarly evaluated expression profiles of CD4^+^CD8^+^ CD69^–^ thymocytes from the mutant A238L transgenic mice were comparable to those of wild type mice. These features, together with the demonstration of (mono-)oligoclonality, suggest a transgene-NFAT-dependent transformation yielding a lymphoma with a phenotype reminiscent of some acute lymphoblastic lymphomas.

## Introduction

Viruses have evolved multiple strategies to manipulate and evade host cell biology and immune responses [1]–[8]. Paradoxically, however, virus host evasion genes potentially provide ready-made tools to explore and manipulate the regulation of these basic cellular processes. We have therefore advanced this concept through construction of a transgenic mouse with T cell restricted expression of a virus inhibitor of transcription, the A238L gene of African Swine Fever Virus (ASFV). One domain of A238L, with homology to IκBα, interacts with p65 of the NF-κB family of transcription factors, thereby inhibiting its activation [9], [10]. Another domain interacts with calcineurin phosphatase (CanPase), (now known as protein phosphatase 3C or PP3C), thus inhibiting activation of NFAT transcription factors [11]–[13]. A mutant A238L (mutA238L) no longer capable of interaction with CanPase, but still inhibiting the activation of p65, has been characterised [12]. A third function of A238L was more recently described and results in inhibition of p65/RelA acetylation and inhibition of protein kinase C-θ-dependent-p300 transcriptional activation [14]–[17]. The p300 and CBP proteins play a key role in transcriptional regulation of a myriad of genes which do not bind directly to the promoters of such genes, but are recruited through interaction with several transcription factors, for example, NF-κB and NFAT [18]–[20].

A number of mice lacking expression of NF-κB or NFAT have been constructed in order to define the roles of these transcription factors. Manipulation of such key intracellular signalling molecules through transgenic expression of a viral host modification gene, however, provides an alternative strategy to explore the role of such proteins. As the transcription factors targeted by the A238L transgene are expected to have pleiotropic effects in a variety of cell types, expression of A238L as a conventional murine transgene *in vivo* might be lethal, or altogether too complicated to analyse. Therefore, expression of the A238L virus transgene was restricted to T cells, as the development and function of these lymphocytes is very well understood, thereby providing an excellent system to explore the impact of the transgene.

The resulting T cell restricted A238L transgenic mice developed transplantable, angiogenic thymic tumours, whose T cells were CD4^+^CD8^+^CD69^−^ (mono-)oligoclonal lymphoblasts, with uncontrolled growth in the thymus and metastasis to both the secondary lymphoid organs (spleen and lymph nodes) and to non-lymphoid tissues, such as kidney, lung and liver. In contrast, the mutA238L transgenic mice were indistinguishable from wild type mice, suggesting that NFAT is essential for the neoplastic transformation in the A238L, T cell restricted transgenic mice.

**Figure 1:**
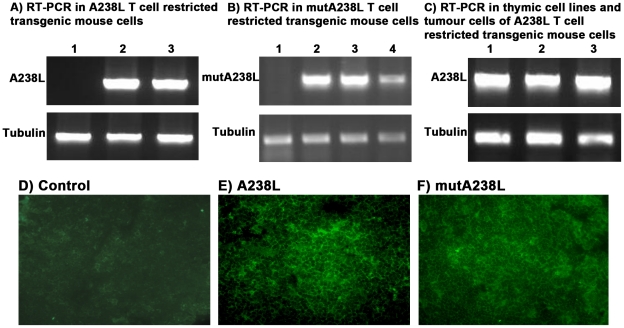
Expression of the A238L transgene confirmed by RT-PCR and by detection of the protein. A) B) RT-PCR for the A238L gene is negative in transgenic splenic B cells after MACS purification (lane 1), and positive in total splenocytes (lane 2) and total thymocytes (lane 3). The upper bands are the A238L PCR product, and the bottom bands are the tubulin controls. C) A similar RT-PCR analysis of two cell lines independently established from the thymuses of two different A238L T cell transgenic mice (lanes 1 and 2), and of a tumour that developed as the result of subcutaneous injection of thymic transgenic cells (lane 3). The upper bands are the A238L PCR product, and the bottom bands are the tubulin controls. Immunofluorescent staining of control (D), A238L (E) and mutA238L (F) T cell restricted transgenic thymus cryostat sections using a rat monoclonal antibody against the HA immunotag upstream the A238L transgene, followed by a goat anti-rat FITC (magnification 40X). The results are representative of at least 5 mice per founder and per genotype.

## Results

### Evaluation of the Number of Copies of the Transgene

The number of copies of the transgene incorporated in the genome of the two A238L founders, as assessed by the Light Cycler technology, was 10 and 30, and the transgene sequence was identical to the published NCBI A238L sequence. The phenotypic analysis presented was performed in two of the four founder mouse lines.

The number of copies of the transgene incorporated in the genome of the mutA238L founder mice, as assessed by the Light Cycler technology, was estimated to vary between 10 and 20. The phenotypic analysis presented was performed in two of the four founder mouse lines.

### Expression of the Transgenes

Expression of the transgenes, A238L and mutA238L, was confirmed at the RNA level by RT-PCR, and at the protein level by western blot and immunofluorescence using an antibody specific for the HA immunotag. Expression of the A238L and mutA238L transgene in purified splenic B cells was either negative or low, in the latter case attributable to contamination of the purified B cells (Figure 1A and 1B). In all the F1 and F2 mice from both founders of each genotype that were analysed, expression of the transgene was confirmed at both protein and RNA levels with transgenic thymus, spleen, lymph nodes, in the organs where there was infiltration of the transgenic cells, and also in the thymic cell lines established from the A238L transgenic mice (Figure 1C). Expression of the A238L and mutA238L transgenic proteins was confirmed by immunostaining of transgenic thymus cryostat sections (Figure 1E and 1F).

**Figure 2:**
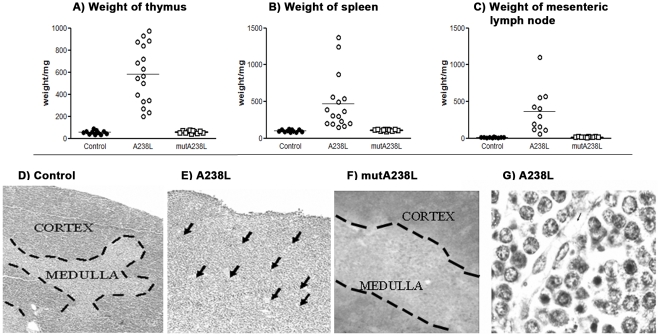
Enlargement and loss of organization of lymphoid organs in A238L T cell transgenic mice. Weight of the Thymus (A), Spleen (B) and Mesenteric Lymph node (C) of control, A238L and mutA238L T cell restricted transgenic mice. Each symbol represents an animal and black and white circles and white squares correspond to 11 control, 17 A238L and 11 mutA238L transgenic mice, respectively. Haematoxylin eosin stained sections of control mouse thymus (D) showing the typical cortex, with densely packed cells clearly differentiated from the medulla with less densely packed cells, and transgenic A238L thymus (E), containing blood vessels (see arrows) and lacking structural organization. The infiltrate of the thymus is accompanied by an increase in vascularisation (see arrows) (magnification 10X). At higher magnification, the transgenic thymus was seen to consist of typical lymphoblasts and significantly more dense apoptotic nuclei than the control thymus (G) (Magnification 40X). Vascularisation of the T cell restricted A238L transgenic thymus is also shown in the photograph (G), (note: blood vessel with its endothelial cell wall and shadows of red cells in the lumen). The results are representative of at least 5 mice per founder and per genotype.

### Clinical Symptoms and Phenotypic Analysis of A238L Transgenic Mice

Both homo and heterozygous A238L transgenic mice developed breathing difficulties, lost body fat and displayed a darker red tone in their eyes, suggesting that they were not oxygenating properly. The onset of these symptoms was variable, occurring between 2–12 months of age. The sick A238L mice always had an enlarged thymus, up to 15–20 times the size of a normal thymus (Figure 2A), and also presented enlarged spleens and lymph nodes (Figures 2B and 2C). There was a dramatic difference in the organisation of the A238L transgenic thymus on the one hand (Figure 2E), and the wild type (Figure 2D) or mutA238L transgenic thymuses on the other (Figure 2F). Most of the cells in the A238L transgenic mice were typical blasts with a prominent nucleolus, peri-nuclear chromatin and with cytological features of lymphoblastic lymphoma cells. There was an increase in cells with dense apoptotic nuclei in the transgenic thymus (Figure 2G). Importantly, the organisation of the mutA238L transgenic thymus was indistinguishable from the control mice.

Increased proliferation in A238L transgenic thymuses was confirmed by both cell cycle analysis of thymocyte cell suspensions using propidium iodide and by incorporation of bromodeoxyuridine (BrdU) *in vivo* (data not shown). Again, cell cycle analysis and incorporation of BrdU *in vivo* yielded similar results in the control and mutA238L transgenic mice.

**Figure 3:**
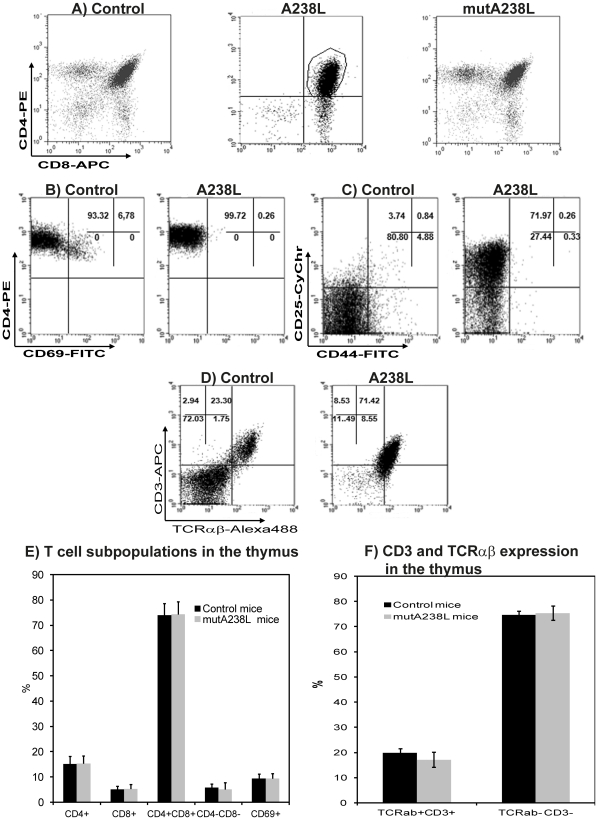
Phenotype of thymocytes from T cell restricted A238L transgenic mice. A) Surface phenotype of thymic cell suspensions of control littermates (left), Tg A238L (middle) and Tg mut A238L mice (right). Cells were stained with antibodies specific for CD4-PE, CD8-APC and CD69-FITC cell surface markers. Results are presented as CD4 versus CD8 (A) and the indicated CD4^+^CD8^+^ cells were then examined for CD69 expression (B, presented as CD4 versus CD69, and graphs E and F). The same cells were also stained with CD4-PE, CD8-APC, CD25-CyChr and CD44-FITC, and the expression of CD25 and CD44 on CD4CD8 double positive cells was determined (C, presented as CD25 versus CD44). The analysis was also performed for splenic and lymph node cell suspensions and the same results were obtained (not shown). The reduced expression of CD3-APC and TCRαβ-Alexa488 on T cell restricted A238L transgenic mouse thymocytes was demonstrated by staining with antibodies specific for CD3 and TCRαβ (D, presented as CD3 versus TCRαβ, graphs E and F). The results are for groups of 5 mice per genotype and are representative of at least three independent experiments.

Detailed phenotypic analysis of the A238L transgenic thymus lymphoblasts revealed the predominant population to be CD4^+^CD8^+^CD69^−^ (Figure 3A and 3B), but with lower levels of expression of the αβ-TCR than the normal control TCR positive thymocytes (Figure 3D). The absence of CD69 expression (Figure 3B) suggests that the A238L transgenic cells were derived from T cells prior to positive selection.

**Figure 4:**
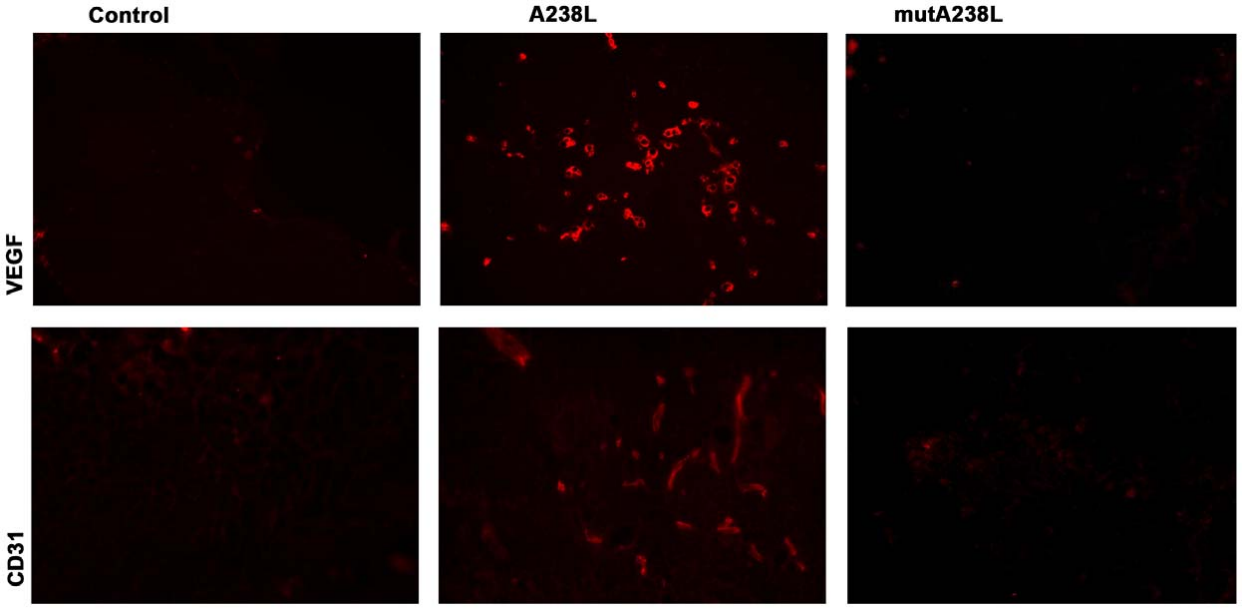
Demonstration of endothelial cells and VEGF synthesis in the thymus of the T cell restricted A238L transgenic mouse. Cryostat sections of thymus of control littermates (left column), A238L Tg (middle column) and mutA238L Tg (right column) mice were stained with antibodies specific for VEGF (upper row) (magnification 20X) and endothelial cells (CD31) (lower row) (magnification 40X). The results are representative of at least 5 mice per founder and per genotype.

**Figure 5:**
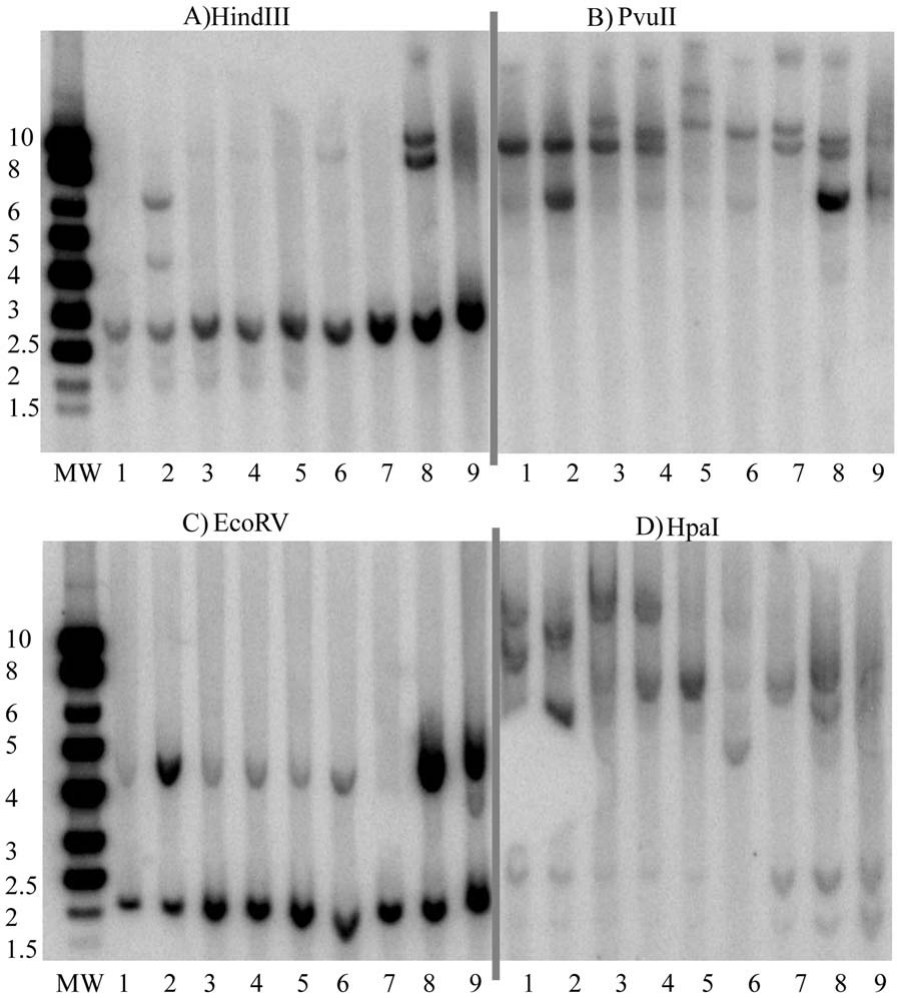
Southern-blot analysis of TCRβ clonality of the thymic transgenic cells in the A238L T cell restricted transgenic mice. 30 µg of DNA from the thymus of transgenic mice (lanes 1 to 6), of transgenic thymic *in vitro* established cell lines (lanes 7 and 8) and of wild type control mice (lane 9) was digested with enzymes A) HindIII, B) PvuII, C) EcoRV and D) HpaI, as indicated on the top of the gels. Lanes 1 to 4 and 5 to 6 are of progeny mice of Founders 1 and 2, respectively. The *in vitro* established cell lines, corresponding to lanes 7 and 8 were derived from Founders 2 and 1, respectively, and do not correspond to the mouse thymus DNA samples analyzed on the gel. Lane MW indicates the molecular weight marker used and the corresponding values are indicated in the text box on its left.

The expression of CD25 and CD44 permits the definition of 4 distinct developmental stages in normal thymocytes prior to positive selection. This analysis of the A238L transgenic animals, clearly demonstrated a very different pattern of A238L transgenic CD4^+^CD8^+^ double positive subpopulations, from that of the control mice. In the latter, the CD4^+^CD8^+^ cells are almost exclusively CD25^–^CD44^–^ (Figure 3C, left), whereas in the A238L transgenic animals the predominant cell (72%) corresponded to CD25^+^CD44^–^ cells and only 27% were CD25^−^CD44^−^ (Figure 3C, right). In young A238L T cell restricted transgenic mice with normal sized thymuses, the subpopulations identified by staining with antibodies against CD4, CD8, CD25 and CD44, against CD4, CD8 and CD69, and against CD3 and TCRαβ, were always the same as that of the wild type control mice. Similarly, staining with Thy-1, which allows evaluation of early haematopoietic differentiation, did not reveal differences in young A238L T cell restricted Tg mice with normal sized thymuses. In contrast, in the tumorigenic Tg mice, there were increased levels of expression of Thy-1 (data not shown). As soon as the thymus was significantly larger, this was accompanied by a clear increase in the population of the CD4^+^CD8^+^CD69^−^ phenotype lymphoblasts.

A similar analysis was performed in the mutA238L transgenic mice and as can be seen the pattern of expression of CD4, CD8 and CD69 is the same as the control mice (Figure 3E), as well as the expression of CD3 and αβ-TCR (Figure 3F) and of CD25 and CD44 (data not shown), which are comparable to the control negative littermates. Similarly, these mice appeared normal, never exhibiting the symptoms described for the A238L transgenic animals.

### Induction of Angiogenesis

The often appearance of blood in the A238L transgenic thymus indicated an angiogenic stimulus, which was confirmed by the presence of visible blood vessels containing red blood cells (Figure 2E), absent in both the mutA238L transgenic and the control mice, and the widespread distribution of anti-CD31 positive endothelial cells and of cells secreting vascular endothelial growth factor (VEGF) (Figure 4, upper images). The actual cell producing the VEGF has not been identified, but it is certainly not an endothelial cell as it was clear by examination of thymus sections counterstained with anti-CD31 (Figure 4, lower images). No such phenotype was detectable in the mutA238L transgenic mice which were always indistinguishable from the control mice (Figure 4, right images).

### Clonality of the T Cells in the Tumours

TCR β-chain rearrangements were defined by Southern blotting of EcoRV, HindIII, HpaI and PvuII digested DNA samples from the thymuses of wild type control mice (Figure 5, lane 9) and progeny mice of both A238L T cell restricted transgenic founders (Figure 5, lanes 1 to 6). In addition, DNA samples from two different thymic cell lines established *in vitro*, each derived from different founder mice, were also digested with the same enzymes (Figure 5, lanes 7 and 8). This Southern-blot analysis uses a TCR β-chain constant region probe to define TCR β-chain rearrangements [21].

Although a given restriction enzyme did not discriminate all eight samples, comparison of the three enzyme digestions (HindIII, HpaI and PvuII in Figure 5) established the unique nature of the restriction enzyme profile, and thus the β-chain V-C integration, of each sample. Thus these transformed cells must have originated from the clonal expansion of a small number of cells, and are therefore of mono or oligoclonal origin.

**Figure 6:**
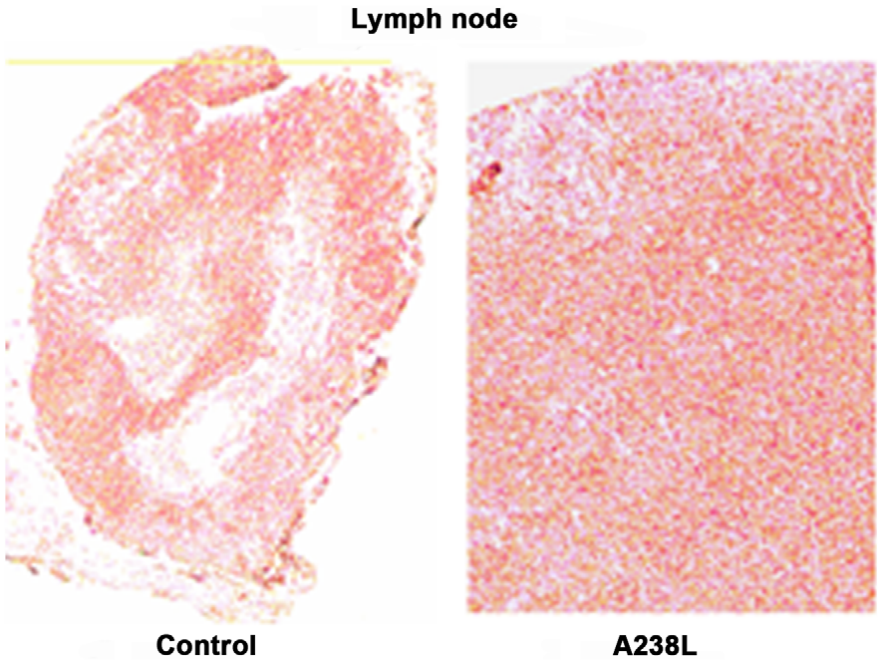
Metastasis of thymocytes from T cell restricted A238L transgenic thymus. The figure shows sections of lymphoid organs stained with Hematoxylin/Eosin (magnification 10 X). There is an exuberant infiltration ofmesenteric lymph nodes, with a corresponding loss of tissue organization. (Not shown: There is also an infiltration of other tissues in areas around arteries and blood vessels, such as kidney, liver and lung and morphology of the infiltrating lymphocytes is similar to that observed in the transgenic thymuses) The results are representative of at least 5 mice per founder and per genotype.

### Metastasis of the Transgenic CD4^+^CD8^+^CD69^−^ Transgenic Thymocyte to Peripheral Lymphoid Organs and Non-lymphoid Tissues

The A238L transgenic mice presented enlarged (up to 15–20 X) spleen and lymph nodes, often with a fibrous consistency, and clearly infiltrated with lymphoblasts of the same CD4^+^CD8^+^CD69^−^, mostly CD25^+^ and CD3^low^, phenotype as in the transgenic thymus (data not shown). In agreement with this, there was a similar incorporation of BrdU *in vivo* in the metastasised T cells of transgenic spleen and lymph nodes (data not shown). In contrast, and as expected, in splenic T cells from control and mutA238L transgenic mice, there was little or no proliferation of either T or B cells. At the same time, however, the B220 positive B cells present in A238L transgenic spleens did not incorporate BrdU, consistent with the T cell restricted expression of the transgene (results not shown).

**Table 1:** Expression analysis of CD4^+^CD8^+^CD69^−^ thymocytes reveals a distinctive pattern of some affected categories of genes for the A238L transgenic mice.

Gene ontology	Number of genes affected/category	
	A238L vs Wt	A238L Vs mutA238L
cell cycle	87	88
cell division	59	59
chromosome, pericentric region	15	15
cyclin-dependent protein kinase regulator activity	12	12
cytokinesis		11
DNA binding		210
DNA replication	43	47
DNA replication initiation	8	9
DNA-dependent ATPase activity	10	11
DNA-directed DNA polymerase activity	10	12
kinetochore	9	212
mitosis	41	43
nuclease activity	22	21
nucleobase, nucleoside, nucleotide and nucleic acidmetabolic process	12	13
nucleotidyltransferase activity	21	20
nucleus	458	464
purine nucleotide biosynthetic process	7	7
replication fork	7	7
ribonuclease H activity	7	6
RNA binding	78	84
single-stranded DNA binding	11	14

As observed in the A238L transgenic thymus, the uncontrolled proliferation of metastasised transgenic CD4^+^CD8^+^CD69^−^ lymphoblasts completely disrupted the histological organization of spleen and lymph nodes (Figure 6 shows only the mesenteric lymph node). No organised B cell follicles or germinal centres were observed. Instead, immunofluorescent staining of transgenic spleen, revealed a homogeneous histology with the B cells randomly scattered in a mass of T cells (photo not shown). The metastasising transgenic CD4^+^CD8^+^CD69^−^ lymphoblastic T cells also colonized non-lymphoid tissue, such as liver, kidney and lungs (data not shown). These organs were fibrous and usually presented a paler colouration, particularly in the case of the kidney and liver. Infiltrating lymphoblasts were of similar morphology to those observed in the transgenic thymuses.

Expression of the transgene in these cellular infiltrates was always confirmed by RT-PCR or western-blot.

**Table 2:** Expression analysis of CD4^+^CD8^+^CD69^−^ thymocytes reveals a distinctive pattern for the A238L transgenic mice.

x Mice/y mice	Number of genes with fold expression ?Tg/Wt ?≥3	P value
A238L/control	1284	0.000001<P<0.00036
A238L/mutA238L	1345	0.000001<P<0.000851
mutA238L/control	0	0.000199<P<0.000878

### The A238L Transgene Impairs Expression of NF-κB and NFAT Downstream Genes

The expression profiles of the main CD4^+^CD8^+^CD69^−^ population of thymic tumour cells present in the thymus of the A238L transgenic mice were compared to the same cell populations from wild type and mutA238L mice. For this, cDNA was prepared from the purified population and hybridized onto GeneChip® Mouse Genome 430A 2.0 Array (Affymetrix). This Chip is a single array representing approximately 14,000 well-characterized mouse genes that can be used to explore biology and disease processes, and in this case, to evaluate the impact of the T cell selective expression of the A238L gene.

The analysis was performed by comparison of the data for the purified transgenic thymic A238L T cells with cells of the similar surface phenotype of the control mice, and considering that only differences equal or greater than the absolute value of 3 are relevant. The same rationale was used to compare the results for the mutA238L and the control mice and for the A238L and the mutA238L mice.

As can be seen on Table 1, the results of the comparison of the A238L and the control and mutA238L are essentially the same in respect of the number of affected genes as well as the category of these genes.

When the results of the purified thymic T cells from the A238L transgenic were compared to those obtained with wild type and mutA238LT cells, a total of 1284 and 1345 genes, respectively, displayed an absolute fold expression difference greater than 3 (Table 2). Interestingly, there was little difference comparing the expression analysis of the mutA238L and wild type T cells.

**Figure 7:**
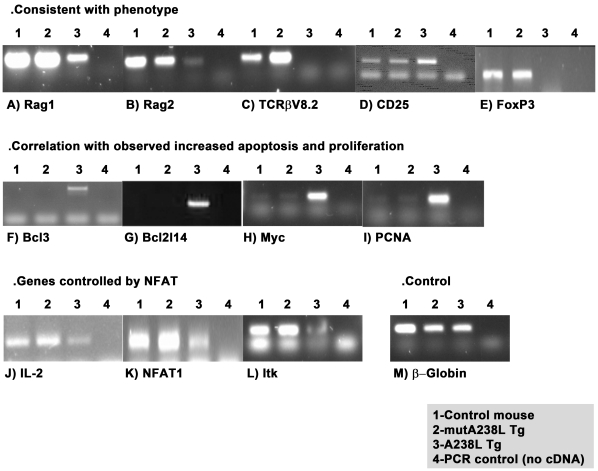
Expression analysis of CD4^+^CD8^+^CD69^−^ thymocytes demonstrates selective effect of the expression of the A238L transgene in the A238L transgenic mice. Semi-quantitative PCR analysis of the cDNA prepared from the mRNA of the purified CD4^+^CD8^+^CD69^−^ thymic cells of A238L and mutA238L transgenic and control mice shows that the A238L transgenic mice display a distinctive pattern of expression for a group of selective genes which correlates to the phenotype of the mice. β-Globin was used as the housekeeping gene. The results presented correspond to a pool of 5 mice per genotype.

The results of the gene chip analysis were then confirmed for selected genes by semiquantitative PCR (Figure 7). The reduced expression of the recombination activating gene 1 (Rag1) and Rag2 genes, whose expression is crucial for the establishment of a T cell receptor diversity is consistent with the existence of a limited T cell repertoire, as detected by flow cytometry analysis (data not shown), and also confirmed by the absence of expression of TCRβ-V8.2 (Figure 7A, 7B and 7C). The high expression of CD25 observed in the A238L transgenic T cells was confirmed by both the array and the PCR results (Figure 7D). Interestingly, the A238L transgenic T cells did not express the forkhead box P3 (FoxP3) transcription factor, a specific marker of both natural and adaptive/induced regulatory T cells [22], whereas there was no such difference between the control and the mutA238L mice (Figure 7E). Whether this difference is a direct or indirect consequence of the expression of the transgene is not clear. There is no doubt, however, of a profound impact of the A238L transgene on the population of T cells so generated.

Three genes (Bcl3, Bcl2114, and Myc) were evaluated by PCR due to their known impact on apoptosis and proliferation, two features observed to be augmented in the A238L T cells, and reflected by their increased expression as seen in the PCR (Figure 7F, 7G and 7H).

Similarly, the PCR analysis of the A238L transgenic T cells confirmed: (1) the exhuberant expression of the Proliferating Cell Nuclear Antigen (PCNA) (Figure 7I), consistent with the increased proliferation of the A238L transgenic thymocytes demonstrated by incorporation of BrdU; (2) the decreased expression of 2 genes (IL-2 and NFAT-1) controlled by the NFAT transcription factor (Figure 7J and 7K) and (3) The reduced expression of the IL-2 inducible T cell Kinase gene (Itk), which is controlled by IL-2 and thus the predicted result (Figure 7L).

## Discussion

This work demonstrates that virus host evasion genes can be used as probes to manipulate the genetic programme of mammalian cells *in vivo*. Thus, T cell restricted transgenic expression of a virus host modification gene (A238L of ASFV) inhibiting transcription mediated by both p65 (NF-κB) and NFAT has resulted in a transformation event at the CD4^+^CD8^+^ stage of T cell development yielding an aggressive (mono-)oligoclonal, angiogenic, metastasising and transplantable CD4^+^CD8^+^CD69^−^ lymphoma. This pattern of expression of CD4 and CD8 DP has been previously described for some human T cell acute lymphoblastic leukaemias [23] and in mice with T cell restricted transgene expression of c-myc [24].

The viral gene selected for this study was the A238L of ASFV, a dual inhibitor of NF-κB and NFAT mediated transcription [9]–[12]. Although in the course of a natural infection with ASFV the cells that are infected are porcine macrophages and not mouse T cells, A238L has been shown to maintain its activity as an inhibitor of NFAT and p65 when the viral protein is expressed in monkey fibroblasts (Vero cells), in human T cells (Jurkat cells) and in mouse macrophages (Raw 264.7 cells) [10], [13], [14], [25]. This non-specific species and tissue impact predicts that the A238L would function at the level of murine T cells, as the transgenic tumour phenotype confirms.

A key question is whether the neoplastic transformation of the A238L transgenic mice results from expression of the transgene or is simply the consequence of its integration site. The available evidence strongly argues in favour of the first alternative. Thus, integration of the A238L transgene into the genome occurs randomly and yet progeny of two distinct founder mice presented the same phenotype. Second, sequencing of the A238L transgene in both founder mice demonstrated the presence of the entire un-mutated sequence. Third, the fact that the only cells whose development is abrogated and disrupted are T cells, is strong evidence that the effect observed is the direct result of selective A238L transgene expression. Fourth, direct proof of transgene expression was demonstrated in transgenic thymus at mRNA (RT-PCR) and protein (fluorescent antibody) levels.

Importantly, a similar transgenic mouse was created, but expressing a point mutated version of the A238L which results in its inability to interact with CanPase, so that this transgene solely inhibits the activation of p65. As described in the Results, these transgenic mice were indistinguishable from the wild type animals. We may therefore conclude that inhibition of activation of NFAT is essential for the observed neoplastic transformation in the A238L transgenic mice, and that inhibition of activation of p65 may not be a key factor in the mechanism of the observed neoplastic transformation.

The analysis of the pattern of expression of these CD4^+^CD8^+^CD69^−^ thymocytes, assessed by array analysis and confirmed by PCR, revealed a series of coherent and relevant information that is consistent with the phenotype presented by the A238L transgenic mice and with the outcome anticipated due to the expression of A238L; that is, inhibition of activation of p65 and inhibition of CanPase with consequent lack of activation of the NFAT proteins. Thus, the array and PCR analysis demonstrated increased expression of the Bcl3, Bcl2l14, Myc and PCNA and decreased expression of NFAT1, IL-2 and Itk.

The Bcl3 gene is a proto-oncogene candidate [26], currently considered to be a molecular marker of anaplastic large cell lymphoma [27] and over-expression of the Bcl2l14 gene has been shown to induce apoptosis in cells [28], [29]. The induction of apoptosis in the A238L transgenic thymus is consistent with the increased expression of Bcl2l14 revealed by the chip and confirmation by PCR analysis. Similarly, Myc has been associated to several forms of cancer and is known to drive cell growth and cell proliferation, up-regulating cyclins [30]. The Proliferating Cell Nuclear Antigen (PCNA) gene codes for a protein that acts as a processivity factor for DNA polymerase δ in eukaryotic cells [31] and its exuberant expression in the A238L transgenic mice confirms the PI and BrdU flow cytometry analysis. As expected, the expression of two genes whose expression is known to be highly controlled by the NFAT family of transcription factors, IL-2 and NFAT1 was dramatically decreased in the A238L transgenic mice. Finally, the IL2-inducible T cell kinase (Itk) gene, codes for an intracellular tyrosine kinase expressed in T cells which plays a major role in T cell proliferation and differentiation, regulating signalling from the T cell receptor (reviewed in [32]). Its expression is induced by IL-2 itself, so it is therefore not surprising that the A238L transgenic tumorigenic T cells displayed a dramatic reduction in its expression, when compared to control and mutA238L transgenic mice. Significantly, the expression analysis of the mutated A238L transgenic mouse thymocytes was comparable to normal thymocytes.

These features, together with the demonstration of (mono-) oligoclonality, suggest a transgene-NFAT-dependent transformation yielding a lymphoma with a phenotype reminiscent of some acute lymphoblastic lymphomas. The detailed molecular mechanism of the transgene induced transformation, like many neoplastic transformations, is still not clear, and the multiple activities of the A238L transgene (see introduction) certainly provide a variety of possibilities for future exploration in the transgenic mouse in the future.

Virus strategies for manipulating transcription provide tools for understanding mechanisms of transcription and may complement the analysis of knock-out mice. Extensive work has been done to elucidate how some families of transcription factors impact on T lymphocyte development and function. Several knock-out mice, both with T cell selective and whole body expression, have been constructed for different NFAT and NF-κB proteins, as well as mice that over express IκB proteins. However, the resulting phenotypes are not always predictable from previous data because it is believed that the outcome depends on the cellular context, that is the cell from which the transcription factor or inhibitor was knocked-out from, or in which the inhibitor protein was over-expressed. Moreover, knocking-out a given NF-κB protein may not be necessarily equivalent to over expressing its respective inhibitor.

There are, however, some correlations between the phenotype of our transgenic mouse and the previously described NF-κB and calcineurin deficient mice. For example, it is known that NFAT4 controls the transition of developing thymocytes from the DP CD4^+^CD8^+^ immature stage to the CD4^+^ or CD8^+^ single positive stage [33], and a tumour suppressor function has also been indicated for NFAT4 in the genesis of T cell lymphomas induced by insertion of the retrovirus SL3-3 [34]. Particularly relevant in this study, NFAT4 KO mice developed tumours with immature CD4^+^CD8^+^ DP thymocytes. Also, deletion of calcineurin Aβ resulted in reduced numbers of SP thymocytes, which demonstrates the involvement of the enzyme in positive selection [35]. The fact that the transformed lymphoblasts of the A238L transgenic mice do not progress from the DP to the SP stages of thymocyte development may therefore correlate with the effect of knocking out NFAT4. Moreover, the A238L transgenic mice also displayed massive lymphadenopaty and splenomegaly, as it has been described for double KO for NFAT1 and NFAT4 mice [36]. On the other hand, mice deficient in individual NF-κB subunits show no intrinsic defect in the establishment of normal population in the T cell lineage [37]–[42]. Again, and interestingly, our mutA238L T cell restricted transgenic mice behaved like this, as they exhibited normal population of T cells and were indistinguishable from the control animals in all of the parameters that we evaluated.

The IκB domain of the A238L molecule is expected to inhibit NF-κB activation. The possible importance of NF-κB in positive and negative selection of T cells is indicated by over expressing the IκBα inhibitor in T cells, which results in a reduction of the proportion of mature TCR^high^ of the αβ-lineage coupled with an unusually high proportion of DP thymocytes [43]. Taking all of the above into consideration, the phenotype exhibited by our A238L T cell restricted transgenic mice could be the result of inactivating NFAT without necessarily inactivating the NF-κB family of transcription factors, this in turn causing a transformation event at the DN stage, perhaps prior to the process of positive selection as evidenced by the absence of expression of CD69 in the thymus. The absence of CD69 expression in the lymphoma suggests that the transformation event took place prior to positive selection. It must be admitted, however, that a possible participation of NF-κB can not be entirely ruled out.

In conclusion, virus host evasion genes may function as tools for genetic manipulation at the level of the whole animal. Elucidation of the molecular events associated with the development of this virus host evasion molecule induced metastasising, angiogenic tumour may clarify some mechanisms of tumorigenesis in general, and in the development of T cell acute lymphoblastic leukaemia in particular. Finally, the phenotype of the T cell restricted A238L transgenic mouse would have been predicted from the recent suggestion for targeting calcineurin activation as a therapeutic intervention in acute T cell lymphoblastic leukaemia [44].

## Materials and Methods

### Mice

FVB/N mice were produced and kept at the IGC (Oeiras, Portugal). The B6xCBA mutA238L founder mice were produced at the Transgenic Facility of Umeå University, Sweden and kept at the IGC (Oeiras, Portugal).

### Ethics Statement

All the animal work was conducted in compliance with the Portuguese and European laws (Portaria 1005/92 and Directive 86/609/EEC, respectively), and following the FELASA recommendations. All animal experiments were performed at the IGC animal house, which is licensed by the portuguese national entity, Direcção Geral de Veterinária (Laboratory permission 520/000/000/2518/99). All efforts were made to minimize animal suffering.

### Gene Amplification and Plasmid Construction

The A238L gene was amplified by PCR from DNA of the non-pathogenic ASFV Ba71v strain using Pfu DNA polymerase, dNTPs 200 µM, primers 1 µM, MgSO_4_ 3 mM, 95°-2′, 30X (95°-1′, 44°-1′, 74°-2′), 74°-5′, and the primers: Up, 5′-CTAGAATTCATGGAACACATGTTTCCA-3′, Low, 5′-CTACTCGAGTTACTTTTCATACTTGTT-3′. A mutated version of A238L, (mutA238L) with mutations introduced in the CanPase binding motif of A238L, yields a protein which no longer interacts with CanPase, and is, thus, incapable of inhibition of CanPase,was provided by Dr Linda Dixon. The mutations were introduced in residues 200 to 213 [12], leaving the ability of mutA238L to inhibit p65 intact.

The gene was cloned as a 5′EcoRI-3′XhoI fragment into the SVA(-) vector, which confers T cell specific expression, with an upstream Influenza haemaglutinin peptide (HA) tag. The expression cassette was released from the bacterial backbone (pBluescript SK-) by digestion with SfiI and microinjected into FVB/N fertilized eggs, which were implanted into pseudo-pregnant foster females. Founder mice for the A238L transgenic were crossed with wild type FVB/N mice to obtain hemizygous mice. Subsequent crossing of hemizygous mice, yielded homozygous animals. The same procedure was used for the founders of the mutA238L B6xCBA founder mice and all the analysis were performed in the FVB/N strain, easily identifiable by the coat white colour. Control animals were littermates without the transgene.

### Sequencing

Sequencing was done using primers derived from the sequence of the SVA(-) plasmid, specifically, upstream and downstream of the EcoRI and XhoI sites, respectively: Up, 5′-TTCTTCCAAAGGTAAGCATAAGAGTCAAAG-3′ and Low, 5′-GTTTCTATCTTTTTTAATTAGAGGAAGGGG-3′. The kit used was BigDye terminator v1.1, Part N° 4336776, Applied Biosystems and the cycling conditions were 96°-1′ and 25X (96°-10″, 50°-5″, 60°-4′). The PCR products were analysed on a 377 DNA Sequencer and 3130xl Genetic Analyser, Applied Biosystems.

### Construction of the Transgenic Mice

In order to achieve selective expression of the transgene in T cells, the A238L virus gene was sub-cloned into a plasmid containing a modified human CD2 promoter which ensures expression of the transgene at the DN stage of T cell development, with minimal expression in B cell lymphocytes and ensures that expression is independent of the integration site and proportional to the number of integrated copies of the transgene [45].

Four A238L transgenic mice, two males and two females, were obtained from a litter of 7 mice after injection of fertilized eggs with an expression cassette containing the A238L transgene and an upstream HA immuno-tag. Upon Southern blot analysis, three of the four mice presented a transgenic DNA fragment of the expected size. One of the two females exhibited a smaller than expected transgene and was discarded, and the second female founder mouse died. The remaining two male founders were crossed with FVB/N females and the F1 mice were bred to yield F2 mice.

For the construction of the mutA238L transgenic mice, two male and two female transgenic mice, were obtained from a litter of 7 mice after injection of fertilized eggs with the expression cassette of the SVA plasmid containing the mutA238L transgene and an upstream HA immuno-tag. Upon Southern blot analysis, all the founder mice presented a transgenic DNA fragment of the expected size, and were crossed with wild type FVB/N mice. The resulting F1 mice were further crossed to yield the F2 generation and only the mice that were of the FVB/N phenotype (white colour) were kept and analysed. The sequence of the transgene integrated into the DNA of the 4 transgenic founder mice was determined, after PCR amplification with Pfu, and was complete and identical to the published NCBI A238L sequence, apart from the point mutations introduced into the CanPase binding sequence [12].

### Mouse Genotyping by Southern Blot and PCR and Sequencing of the Transgene

Samples of genomic mouse DNA (10 µg) isolated from tail biopsies by standard methods were digested with EcoRI and XhoI and electrophoresed on a 0.8% agarose gel. Treatment of the gel, transfer to the membrane Hybond-N^+^ and hybridization were done according to the instructions of Hybond-N^+^ (Amersham, U.K.). The 718 bp A238L probe was labelled with α^32^P-dCTP using the Random Primers DNA Labelling System, Gibco, according to the manufacturer’s instructions. Unincorporated probe was removed using G-50 Sephadex Quick Spin Columns, Gibco. The results were also assessed by PCR, as described above.

Mice were then routinely genotyped by PCR using DNA from tail biopsies obtained at the time of weaning (3 weeks old). Tails were digested overnight with Proteinase K (100 µg/ml) at 56°C and then DNA was precipitated with isopropanol (0.7 volumes) at room temperature.

The primers described for sequencing the DNA plasmid construct were also used to sequence the transgene in the genomic DNA of the 2 founder transgenic mice of each genotype, after amplification with Pfu DNA Polymerase as previously described.

### Determination of the Number of Copies of the Transgene

The number of copies of the transgene was evaluated by the Real Time PCR technique using LightCycler FastStart DNA Master SYBR Green I (Roche).

The amount of amplified A238L transgene (primers Up, 5′-ATGGAACACATGTTTCCAGAAAGG-3′, Low, 5′-TTACTTTCCATACTTGTTCAGTTT-3′) was normalized against the amount of amplified mouse β-Globin (primers Up, 5′-CCAATCTGCTCACACAGGATAGAG-3′, Low, 5′-CCTTGAGGCTGTCCAAGTGATTCA-3′). Conditions were MgCl_2_ 4 mM, primers 200 nM. Program for A238L: pre-incubation 95°-10′, amplification 45X (95°-10′, 70°-5″, 72°-29″), Melting Curve Analysis (65°-15″); program for β-Globin: pre-incubation 95°-10′, amplification 45X (95°-10′, 70°-5″, 72°-20″), Melting Curve Analysis (65°-15″). Analysis was performed with the Roche supplied software and calculations on the amount of amplified product were based on the determination of the second derivative maximum and on an arithmetic adjustment of the baseline.

### Demonstration of Transgene Expression by RT-PCR

Total RNA was extracted from tissue homogenates of thymus, spleen and liver from transgenic and control littermates using Trizol Reagent (Sigma). Samples of RNA were digested with DNaseI (Invitrogen) and cDNA synthesis was performed with MMLV-Reverse Transcriptase (Invitrogen), according to the manufacturer’s instructions. Detection of the A238L gene in the cDNA by PCR was performed as above, but using Taq DNA Polymerase, MgCl_2_ 2 mM and performing the extension at 72°C. As a qualitative and semi-quantitative control, mouse tubulin was amplified using Taq DNA Polymerase with dNTPs 200 µM, primers 1 µM, 1.5 mM and primers were: Up, 5′-ATGCGTGAGTGCATCTCCATCCACGT-3′ and Low, 5′-TTAGTATTCCTCTCCTTCTTCCTCAC-3′ and cycling conditions were 94°-5′, 30X (94°-1′, 55-1′, 72°-2′), 72°-10′.

### Flow Cytometry Analysis of Lymphocyte Surface Proteins

Cell suspensions were incubated with titrated concentrations of the antibodies used, washed in PBS containing 2% FCS and 0.02% sodium azide and subsequently analyzed on a FACScalibur. All rat monoclonal antibodies (CD3-APC, CD4-PE, CD8-APC, CD25-PE, CD25-biotin, CD44-FITC, CD69-FITC and TCRαβ-Alexa488, Streptavidin-CyChrome) were purchased from BD Pharmingen (San Diego, CA). Except for the four colour stainings, Propidium Iodide (PI) was always used to exclude dead cells from the analysis that was performed using the FlowJo software (Tree Star, Inc.).

### Analysis of T Cell Clonality by Southern-blot

Thymus DNA samples were prepared as described for tail DNA. Thymic cell lines were lysed in White Cell Lysis Buffer (WCLB, 3 ml/50×10^6^ cells) (10 mM Tris- HC1 (pH 7.6), 10 mM EDTA (pH 8.0), 50 mM NaCl) and incubated overnight at 56°C with 0.165% (v/v) and Proteinase K (165 µg/ml), and extracted twice with phenol-chloroform-isoamyl alcohol and once with chloroform-isoamyl alcohol. After adding NaCl to 0.1 M the DNA was precipitated with isopropanol (0.6 vol).

30 µg of thymus DNA of wild type and transgenic mice were digested O/N with EcoRV, HindIII, HpaI and PvuII and separated on a 0.7% agarose gel. Treatment of the gel, transfer to the membrane Hybond-N^+^ and hybridization were done according to the instructions of Hybond-N^+^ (Amersham, U.K.). The 454 bp TCR β constant region probe [46] (provided by Dimitris Kioussis, National Institute for Medical Research, U.K.) was labelled with α^32^P-dCTP using the Random Primers DNA Labelling System, Gibco, according to the manufacturer’s instructions. Unincorporated probe was removed using G-50 Sephadex Quick Spin Columns, (Gibco).

### Splenic B Cell Purification with MACS LS Columns and Streptavidin Microbeads

Splenic cell suspensions were depleted of erythrocytes by resuspending in ammonium chloride 0.155 M, potassium hydrogenocarbonate 0.01 M, pH 7.2. After 3 washes in PBS-2% FCS, the cells were labelled with anti-B220-biotin (BD Pharmingen, San Diego, CA), incubated with Streptavidin Microbeads and then purified with MACS LS Columns (Miltenyi Biotec, CA). At least 95% of the recovered cells were B cells.

### Histology and Immunofluorescence

Tissues were fixed in Bouin’s solution (Sigma), embedded in paraffin and stained with hematoxylin and eosin.

For immunofluorescence, tissue samples were frozen in tissue-tek O.C.T. compound (Sakura Finetechnical), and 8 µm cryostat sections were fixed (absolute ethanol, 5′-room temp, followed by acetone, 5′-room temp) and air dried. Rat anti-mouse CD-31 (mAb) and Goat anti-rat (IgG and IgM) Alexa 568 were purchased from BD Pharmingen (San Diego, CA) and from Molecular Probes, respectively. Biotinylated anti-murine VEGF (polyclonal Ab) and Streptavidin-Texas Red were purchased from Peprotech EC and SouthernBiotech, respectively. AnnexinV-Alexa 567 was purchased from Invitrogen, and FITC-Rat monoclonal anti-HA was purchased from Roche.

### Expression Profiles of CD4^+^CD8^+^CD69^−^ Purified Thymocytes

Thymic cell suspensions of Wt, mutA238L and A238L mice (pools of 5 mice) were incubated with titrated concentrations of the antibodies used, washed in PBS containing 2% FCS and subsequently analyzed and purified on a FACSAria cytometer (BD), gating on the thymic cell population that was CD4^+^CD8^+^CD69^−^. All rat monoclonal antibodies (CD4-PE, CD8-APC, CD69-FITC) were purchased from BD Pharmingen (San Diego, CA). The purity of the population was verified by flow cytometry in a FACScalibur cytometer (BD) and was always greater than 98%.

Total RNA from the purified cell population was extracted with Mini RNeasy Kit (QIAGEN), digested with DNaseI (Invitrogen) and cDNA synthesis was performed with the 3 `IVT Express kit (Affymetrix), according to the manufacturer’s instructions. The cRNA was hybridized into GeneChip® Mouse Genome 430A 2.0 Array (Affymetrix), according to the manufacturer’s instructions. This analysis was done once (results are available in the GEO database as follows: GSE34048-Transgenic Expression of A238L, a Virus Host Modification Protein in mouse T cells; GSM840978-mouse CD4^+^CD8^+^CD69^−^ thymocytes purified Wt; GSM840979-mouse CD4^+^CD8^+^CD69^−^ thymocytes purified A238L; GSM840980-mouse CD4^+^CD8^+^CD69^−^ thymocytes purified mutA238L).

The results for the A238L mice results were normalized against the Wt and the mutA238L and only values with more than a 3 fold difference were considered. As a comparative control study, the mutA238L mice results were also normalized against the Wt mice.

The microarray results were confirmed by conventional semi-quantitative PCR using primers designed between adjacent exons to discriminate cDNA from contaminating undigested genomic DNA that might be present and using β-globin as the control housekeeping gene. Amplification to detect the selected genes in the cDNA was performed using Taq DNA Polymerase with dNTPs 200 µM, primers 1 µM, MgCl_2_ 2 mM and performing the extension at 72°C. The PCR amplification program consisted of 20–25 cycles, to ensure that saturation was not reached and that the differences detected in the microarray would be demonstrated in the visualization of the amplified fragments with ethidium bromide in a 0.8% standard agarose gel.

Primers and size of amplicon for the selected genes were: β-globin PCR product 500 bp Up 5′-ccaatctgctcacacaggatagag-3′, Low 5′-ccttgaggctgtccaagtgattca-3′; FoxP3-PCR product 331 bp, Up 5′ ctgcctacagtgcccctagtcat 3′, Low 5′ ctgtctttcctgggtgtacccga 3′; IL2-PCR product 400 bp, Up 5′ atcctgtgtc acattgacac 3′, Low 5′ gcactcaaatgtgttgtcag 3′; Rag1-PCR product 400 bp, Up 5′ ccgtctaccctgagcttcagtt 3′, Low 5′ ctttgggttttagcgtccacgg 3′; Rag2-PCR product 382 bp, Up 5′ tccctgcagatggtaacagt 3′, Low 5′ agtctttctctgtgcaacgg 3′; Bcl3-PCR product 557 bp, Up 5′ tgatgcccatttactctacccc 3′, Low 5′ cataattgcgaacctccaggtc 3′; Bcl2l14-PCR product 316 bp, Up 5′ tttgttggattccaaagtggcttg 3′, Low 5′ aacaggtctgtgatggtcttga 3′; NFAT1-PCR product 375 bp, Up 5′ ctccacggctacatggagaac 3′, Low 5′ agcactcgatggggttggacg 3′; ItK-PCR product 443 bp, Up 5′cgttgaagggctccattgaac 3′, Low 5′ cattcttggatgggtcgtagg 3′; Tcrbeta V8.2-PCR product 408 bp, Up 5′GACATACAAATGCACCTATGCG 3′, Low 5′ TTAGGCCTCTTCCAGAAGTAGA 3′; Myc-PCR product 370 bp, Up 5′aactatgacctcgactacgact 3′, Low 5′ tccacatacagtcctggatgat 3′; CD25- PCR product 329 bp, Up 5′ cccatgacaaatcgagaaagca 3′, Low 5′cagaaatcggtggtgttctctt 3′; proliferating cell nuclear antigen (PCNA)- PCR product 320 bp, Up 5′ acgtctcctt ggtacagctt 3′, Low 5′ gtctcggcatatacgtgcaa 3′.
